# YwdL in *Bacillus cereus*: Its Role in Germination and Exosporium Structure

**DOI:** 10.1371/journal.pone.0023801

**Published:** 2011-08-24

**Authors:** Cassandra Terry, Andrew Shepherd, David S. Radford, Anne Moir, Per A. Bullough

**Affiliations:** Krebs Institute for Biomolecular Research, Department of Molecular Biology and Biotechnology, University of Sheffield, Firth Court, Western Bank, Sheffield, United Kingdom; University of Connecticut, United States of America

## Abstract

In members of the *Bacillus cereus* group the outermost layer of the spore is the exosporium, which interacts with hosts and the environment. Efforts have been made to identify proteins of the exosporium but only a few have so far been characterised and their role in determining spore architecture and spore function is still poorly understood. We have characterised the exosporium protein, YwdL. Δ*ywdL* spores have a more fragile exosporium, subject to damage on repeated freeze-thawing, although there is no evidence of altered resistance properties, and coats appear intact. Immunogold labelling and Western blotting with anti-YwdL antibodies identified YwdL to be located exclusively on the inner surface of the exosporium of *B. cereus* and *B. thuringiensis*. We conclude that YwdL is important for formation of a robust exosporium but is not required to maintain the crystalline assembly within the basal layer or for attachment of the hairy nap structure. Δ*ywdL* spores are unable to germinate in response to CaDPA, and have altered germination properties, a phenotype that confirms the expected defect in localization of the cortex lytic enzyme CwlJ in the coat.

## Introduction

*Bacillus anthracis*, and its close relatives *Bacillus cereus* and *Bacillus thuringiensis* of the *B. cereus* group are able to form endospores (spores) upon nutrient exhaustion. The proteinaceous spore coat layers have a major role in protecting the spore from various harmful substances [1], [2]. In the *B. cereus* group an additional outer layer, the exosporium, forms at the same time as the coat layers [3]. The exosporium is the first point of contact with the host and is the barrier between the spore and its environment. It is composed of ∼52% protein, lipids and carbohydrate [4], and contains at least one paracrystalline layer. The outermost paracrystalline layer, called the basal layer, is found in all preparations from *B. cereus sensu lato* strains [5]–[8]. Attached to the basal layer is the BclA-containing hairy nap. 3D structures of various exosporium paracrystalline layers from *B. cereus* and *B. thuringiensis* have recently been determined using electron crystallography [5]. The structure of the type II crystal which makes up the basal layer [5] suggests that the exosporium can have a protective role, acting as a semi-permeable layer. The basal layer has arrays of crown-like structures that may also act as a matrix for the binding or adsorption of other proteins and a scaffold to which the hairy nap attaches. A number of proteins have been identified in the exosporium [9]–[12] of which a limited number have been characterized [13]–[20]. The spatial locations or structural roles of these proteins within the exosporium remain largely unknown; the one exception is BclA, which is known to make up most of the hairy nap attached to the crystalline basal layer and covering the outer surface of the spore in *B. anthracis* [21]. We have been endeavoring to characterise the structural role of other proteins; one approach is to observe the effect of protein deletions on structure and another is to attempt to localise proteins by specific labelling. In this paper we adopt both these approaches to characterise the role of YwdL, which has previously been identified as an exosporium protein in *B. anthracis* [9], [22].

Spores of *Bacillus* species possessing an exosporium were shown to be more hydrophobic than those without during hexadecane-aqueous partition experiments [23]. A reduction in partition into hexadecane has previously been used to enrich for exosporium mutants of *B. cereus* [13]. One of these mutants has a transposon insertion in the *ywdL* gene. The YwdL protein (accession number ZP_04320586; named GerQ in *B. subtilis*, but not in *B. cereus*, as a germinant receptor already had this designation) was identified as a spore coat component essential for the retention of the spore germination cortex-lytic enzyme CwlJ in the spore coat of *B. subtilis* [24]. YwdL has over 50% amino acid identity in all *Bacilli*, with 87% identity across members of *Bacillus cereus sensu lato* and it has been identified (listed as BA5641) as one of the most abundant proteins in exosporium fractions in *B. anthracis* [9] hence it appeared to be a potentially important structural protein of the exosporium.

Here we report on the association of the YwdL protein with the exosporium, the importance of YwdL for the structural integrity of the exosporium and show that it is required for CaDPA (calcium dipicolinate) mediated germination.

## Results

### Inactivation of the *B. cereus ywdL* gene

A Tn917-LTV1 transposon was used to randomly introduce mutations into the genome of *Bacillus cereus* ATCC 10876 as previously described [13]. Transposon insertion mutant libraries were enriched for mutants with spores of reduced hydrophobicity that transferred less efficiently to the hydrocarbon layer during serial partition into hexadecane, and fresh spore preparations were checked by microscopy. Spores of mutant strain AM1660 retained exosporium, suggesting possible changes in spore surface properties. Following inverse PCR of a HaeIII digest of AM1660 DNA, sequence data obtained using a transposon-derived primer provided 152 bp of flanking sequence after the point of insertion in this mutant. The transposon had inserted into *ywdL*, the second gene of a bicistronic operon containing homologues of the *B. subtilis* genes *cwlJ* and *ywdL* (*gerQ*) The transposon insertion was transferred into a fresh background by generalized transduction using phage CP51ts [13], and the phenotype confirmed.

### Mutant spores have a fragile exosporium

**Figure 1:**
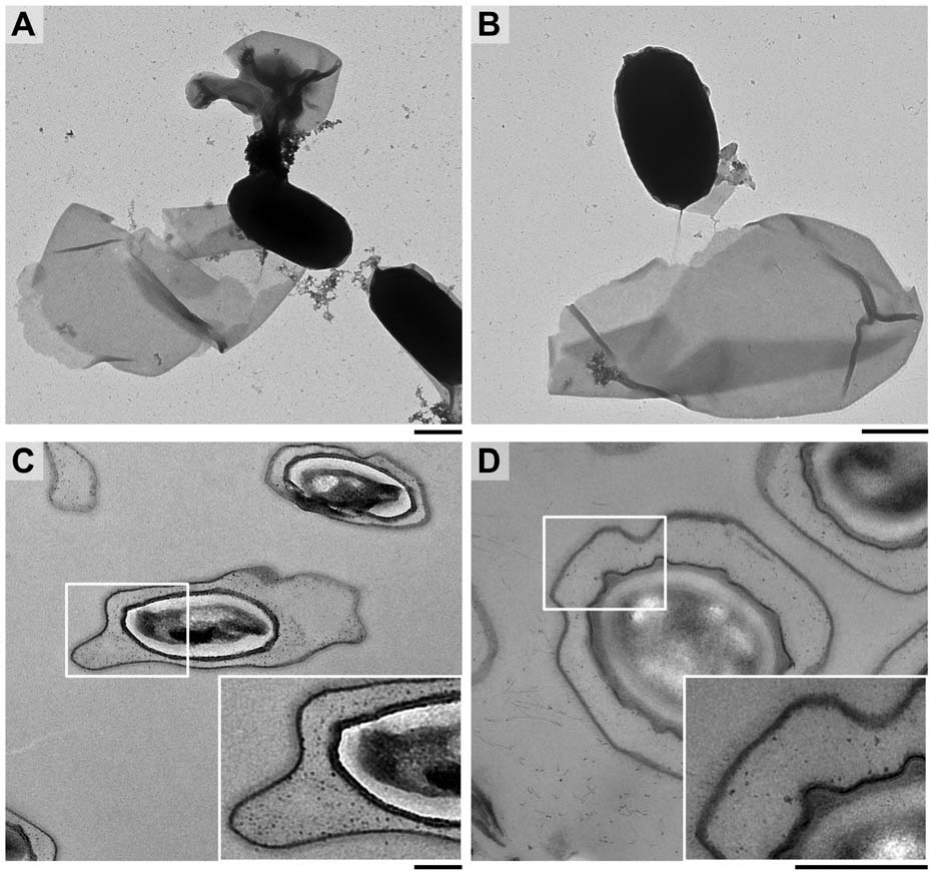
Electron micrographs of Δ*ywdL* and wild-type spores. (A) Negatively stained Δ*ywdL* spores frequently have a fragile exosporium that is no longer intact or (B) is completely detached from the rest of the spore. Spore sections (C) show more electron dense deposits visible in the interspace region in Δ*ywdL* spores compared to wild-type (D). Scale bars are 0.5 µm corresponding to the full size images in (A–D).

Crystal violet staining demonstrated that an exosporium was still present in *B. cereus ywdL::Tn917LTV1* (Δ*ywdL*) spores. *B. cereus* Δ*ywdL* spores sporulated at similar rates to wild-type with a comparable overall spore yield. Electron microscopy revealed overall spore dimensions to be similar to wild-type. However, about 10% of spores had a loosely attached or damaged exosporium (Figure 1A) or in some cases a completely detached exosporium with free exosporium fragments clearly observed on the grid (Figure 1B). Upon freeze-thawing the percentage of spores with abnormal exosporia increased to over 20%. Where the exosporium was still loosely attached to the rest of the spore, it appeared to be attached at only one of the poles. In a spore preparation of the wild-type strain, treated in the same way, fewer than 1% of spores had defective (damaged or missing) exosporia, suggesting that the exosporium is more fragile and loosely attached in Δ*ywdL* spores. Electron microscopy of sectioned spores suggested that there was no major structural defect in the mutant spores, although there was a consistent increase in the amount of particulate material seen in the interspace region between coat and exosporium (Figure 1C and D).

### Resistance and hydrophobic properties of spores

Spore coats are important for protecting the spore from organic solvents and lysozyme [2]. Resistance to wet heat (10 min at 80°C, and 90°C) and ethanol was similar in spores of the Δ*ywdL* and parental strains (Table 1). The spores did not show any loss of OD on incubation with 250 µg ml^−1^ lysozyme for 1 hour, suggesting that the mutation does not alter spore coat structure sufficiently to affect resistance properties. The partition of Δ*ywdL* mutant spores into hexadecane was similar to that of wild-type, suggesting no change in the hydrophobicity of the spore surface of the average spore (Table 1), despite its isolation from enrichment for less hydrophobic spores. This experiment was carried out on fresh (unfrozen) spores, where the exosporium was intact in ∼90% of spores, whereas the library of mutant spores that were initially screened for their partition into hexadecane had been frozen and thawed.

**Table 1:** Spore properties of the ΔywdL mutant in comparison to wild-type.

Spore Properties	Wild-Type	Δ*ywdL*
% resistant to 80°C, 10 min	80±17	72±15
% resistant to 90°C, 10 min	23±7	21±6
% resistant to ethanol	63±9	67±9
% partition into hexadecane	90±2	87±2

Wild-type and Δ*ywdL* spores were tested for their resistance to temperature, ethanol and partition into hexadecane. Tests were carried out three times on each of two independent spore preparations for each strain, and the data averaged.

### YwdL is detected in the exosporium of *B. cereus* and *B. thuringiensis* by immunogold labelling

**Figure 2:**
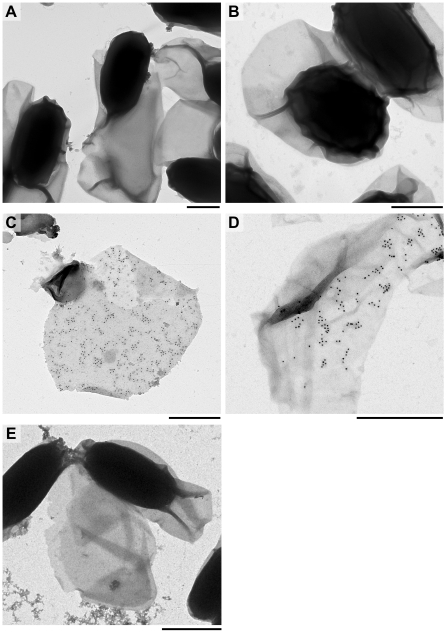
Immunogold labelling of the exosporium with polyclonal anti-YwdL IgG and a secondary antibody conjugated to 10 nm gold. (A) *B. cereus* Δ*ywdL* whole spores (B) *B. cereus* wild-type whole spores, (C) *B. cereus* wild-type exosporium and (D) *B. thuringiensis kurstaki* 4D11 exosporium. (E) *B. cereus* Δ*ywdL* spores and fragmented exosporium. Samples were stained with uranyl formate and analysed by TEM. Scale bars are 0.5 µm.

Water-washed whole spores of *B. cereus* ATCC 10876, *B. thuringiensis kurstaki* 4D11 and *B. cereus ΔywdL* were incubated with anti-YwdL antibody, which was then detected with a gold-labelled secondary antibody (Figure 2). No binding of gold particles was observed in controls using antibodies raised against non-spore associated proteins (data not shown), or to Δ*ywdL* spores with broken exosporium or exosporium fragments when similarly-washed (Figure 2A and E respectively), confirming the specificity of labelling. Notably, wild-type spores with intact exosporium did not bind the antibody; less than three gold particles were visible attached to each such spore of *B. cereus* (Figure 2B) or *B. thuringiensis* 4D11 (data not shown). Extensive binding of gold particles was only visible on exosporium fragments or on loosely attached exosporium pieces of wild-type spores (Figure 2C and D respectively). Therefore YwdL is associated with the exosporium of *B. cereus* and *B. thuringiensis*, and appears to bind antibody only at the inner surface of this layer.

### YwdL is loosely associated with the exosporium

**Figure 3:**
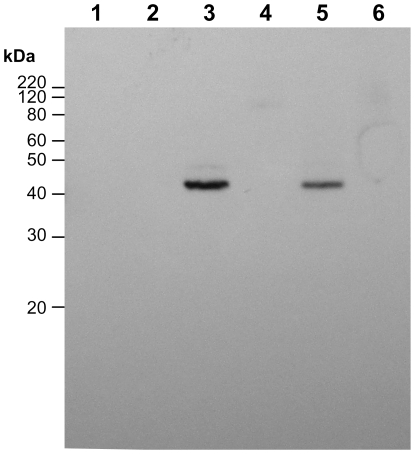
Detection of YwdL in unwashed and fully washed exosporium using Western blotting. An anti-YwdL antibody was used to locate YwdL in unwashed and fully washed *B. cereus* ATCC 10876 and *B. thuringiensis kurstaki* 4d11 exosporium. Lanes 1 and 2 contain unwashed and washed *ΔywdL* exosporium, lane 3 and 4 unwashed and fully washed *B. cereus* exosporium and lanes 5 and 6 unwashed and washed *B. thuringiensis* exosporium respectively.

Proteins were extracted from exosporium fragments that had been released from spores by passage through a French press and then purified away from spores on a density gradient, and probed in Western blots using anti-YwdL antibody (Figure 3). No bands were observed in exosporium extracts from the Δ*ywdL* strain, confirming the specificity of the antibody. A major YwdL band was detected in extracts of unwashed exosporium fragments from both *B. cereus* and *B. thuringiensis* (Figure 3, lanes 3 and 5), at an apparent molecular weight of 43 kDa, although no protein was detected at the predicted monomeric molecular weight of 16 kDa. This confirms the conclusions from immunogold-labelling experiments (Figure 2) that demonstrate the presence of YwdL in the exosporium of *B. cereus* and *B. thuringiensis*. However, after washing fragments with buffers containing 1 M salt and 0.1% SDS to remove loosely associated proteins (whilst maintaining the paracrystalline lattice), this 43 kDa YwdL species was not detected in wild-type exosporium (Figure 3 lanes 4 and 6), suggesting that the YwdL protein is not tightly associated with the exosporium.

### YwdL does not have a major structural role in the exosporium basal layer, or in its assembly

**Figure 4:**
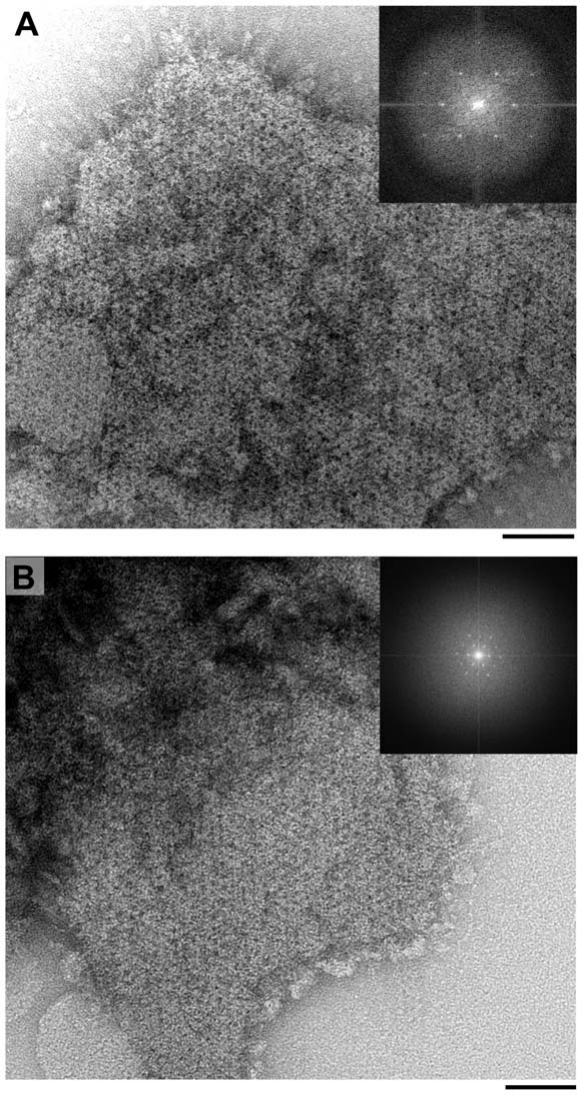
Analysis of ΔywdL fragments by TEM. Isolated exosporium fragments from (A) B. cereus wild-type and (B) ΔywdL were negatively stained and analysed by TEM. Both preparations appear similar. The hairy nap can be seen in both strains protruding out from the hexagonal basal layer in both crystal fragments shown. Fourier transforms (top right corner) were taken from the whole crystal image or from a smaller boxed crystalline area in A and B respectively. First order spots are at a spatial frequency of ∼1/75 Å-1. Scale bars are 50 nm.

Salt and detergent-washed exosporium fragments were viewed by negative stain transmission electron microscopy (TEM). The exosporium crystals of *B. cereus* wild-type and the Δ*ywdL* mutant were indistinguishable (Figure 4). Both have the same unit cell dimensions (*a*≈*b*≈80 Å) with a hexagonal repeating lattice that is clearly visible in both strains. Fragments were of comparable size and the hairy nap was visible in many of the exosporium fragments. Unwashed exosporium fragments were also analysed and appeared to have the same overall morphology in both strains (data not shown).

**Figure 5:**
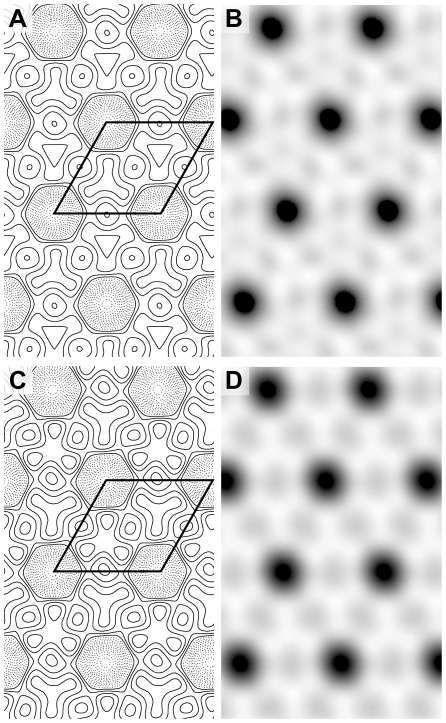
Contour and greyscale projection maps of negatively stained wild-type and ΔywdL exosporium crystals. Each map is the average of 5 independent crystal images taken from the respective exosporium preparation showing (A, B) B. cereus wild-type and (C, D) ΔywdL exosporium crystals. p3 symmetry-averaging has been applied in both cases. One unit cell of edge of 80 Å is outlined. Solid contours (A, C) represent densities below average (protein) whilst dashed contours represent densities above average (stain). White regions (B, D) represent stain-excluding density (protein) whilst black areas represent stain-accumulating regions.

Fourier amplitudes and phases from five different images of wild-type and Δ*ywdL* exosporium respectively were averaged and projection maps calculated (Figure 5). Unwashed exosporium fragments from *B. cereus* wild-type containing YwdL and fully washed preparations devoid of YwdL from *B. cereus* Δ*ywdL* were used for comparison. No significant differences are visible between the two projection structures (Figure 5), with both showing similar density distributions and the same lattice of rings surrounding a large stain-filled core. Both show structures typical of the crystal type II described by Ball *et al.* thought to represent the outermost basal layer previously observed in this strain [5]. This was the only crystal form observed in exosporium preparations from both wild-type and the Δ*ywdL* strain. These results suggest firstly that YwdL does not contribute significant mass to the crystalline components of the exosporium basal layer and secondly that YwdL is not required as part of any assembly machinery for the crystalline basal layer.

### YwdL is not required for BclA localization

**Figure 6:**
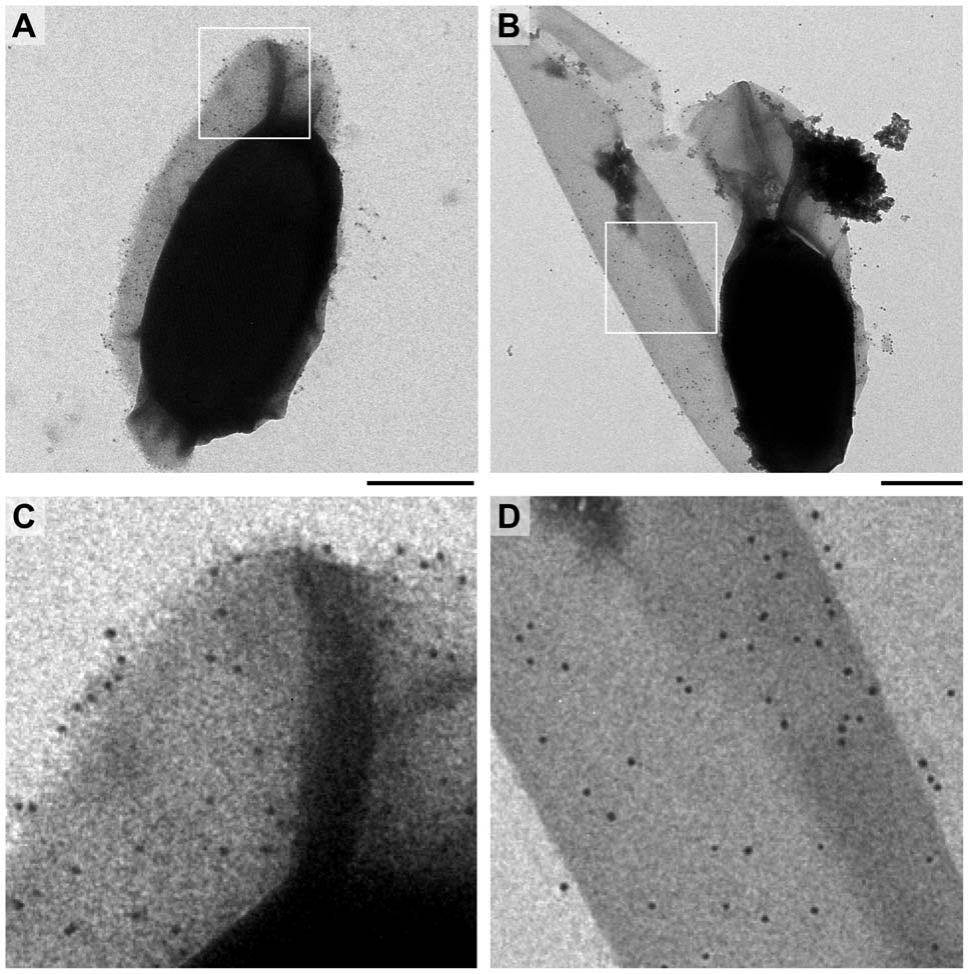
Analysing BclA content of wild-type and ΔywdL spores using immunogold labelling. Whole (A) B. cereus ATCC 10876 wild-type and (B) ΔywdL spores were immunogold labelled using anti-BclA antibodies and a secondary antibody conjugated to 10 nm gold particles. Scale bars are 0.5 µm. Note the fragile exosporium in Figure B. Figure (C) and (D) represent zoomed in areas of (A) and (B) respectively. Scale bars are 0.5 µm.

**Figure 7:**
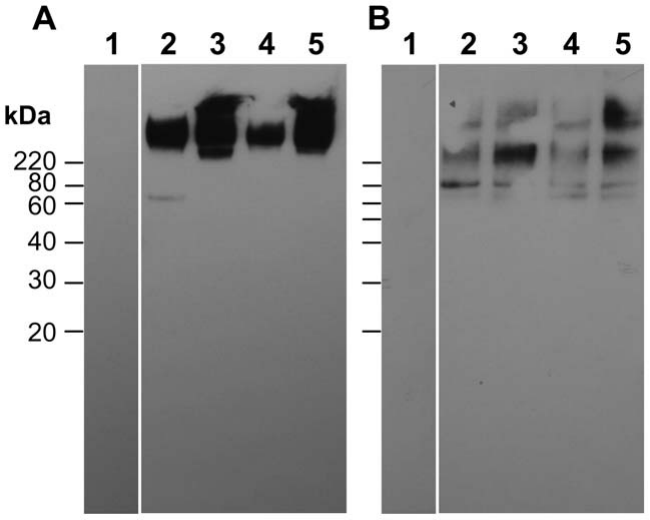
Analysis of the glycoprotein content of wild-type and Δ*ywdL* exosporium. (A) Western blot analysis with anti-BclA antibodies. Lane 1 shows negative control (vegetative cells), lane 2 and 3 unwashed and fully washed *B. cereus* wild-type exosporium and lanes 4 and 5 show unwashed and fully washed Δ*ywdL* exosporium respectively. (B) Total glycoprotein analysis of wild-type and Δ*ywdL* exosporium samples. Lane 1 shows negative control (sodium metaperiodate replaced with 100 mM acetate buffer), lane 2 and 3 unwashed and fully washed wild-type exosporium and lanes 4 and 5 show unwashed and fully washed Δ*ywdL* exosporium respectively. Samples in (B) were extracted under harsher conditions, using solubilisation buffer (see Materials and Methods).

Immunogold labelling with anti-BclA antibodies was performed to check if the distribution and quantity of BclA was affected by disruption of the *ywdL* gene. The extent of gold particle binding was similar for both wild-type and Δ*ywdL* water washed spores (Figure 6). Both strains had >100 gold particles distributed over the entire spore surface, indicating no measurable difference in BclA distribution. Western blotting with the same anti-BclA antibody was performed on isolated exosporium fragments. BclA was detected as a high molecular weight band >220 kDa in unwashed and washed exosporium from wild-type and Δ*ywdL* spores (Figure 7A) as previously described for this strain [16]. A small amount of BclA was detected as a 65 kDa band in unwashed exosporium. Comparable amounts of BclA were present in the exosporium of Δ*ywdL* and wild-type (Figure 7A).

Other glycoproteins with a collagen-like region are likely to be present in the exosporium – for example, ExsJ, found as a major glycoprotein in *B. cereus* exosporium [12]. To investigate whether YwdL is required for the correct incorporation of other glycoproteins, SDS-PAGE-separated proteins from exosporium samples were analysed for total glycoprotein. Proteins were extracted by heating at 90°C in solubilisation buffer to ensure maximum disruption of any protein complexes. The exosporium from the Δ*ywdL* mutant has the same glycoprotein profile as wild-type (Figure 7B). The BclA and total glycoprotein content in exosporium samples is unaffected by washing with salt and detergent buffers.

### Protein composition of the exosporium

**Figure 8:**
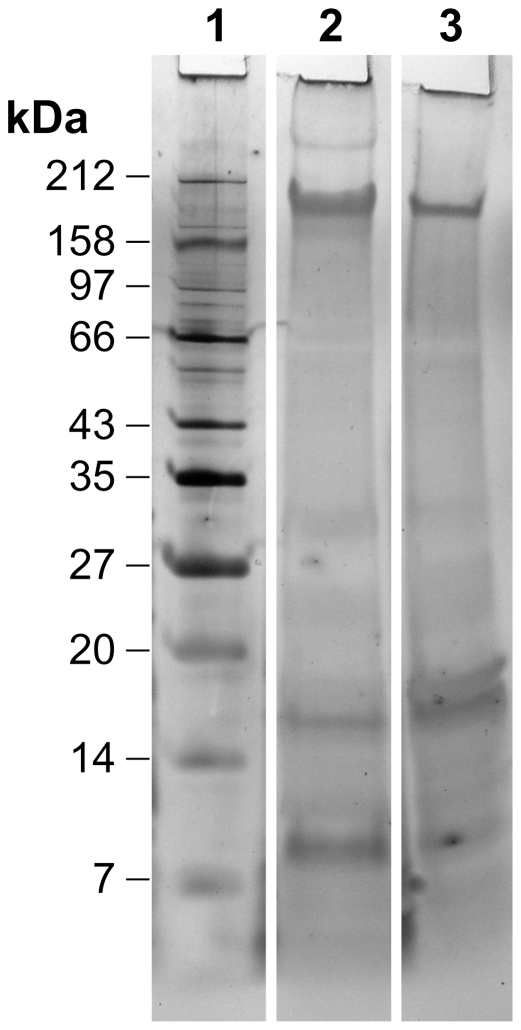
Silver stain PAGE of exosporium of Δ*ywdL* compared to wild-type. Lane 1 corresponds to molecular weight protein standards. Lane 2 shows fully washed *B. cereus* wild-type exosporium and lane 3 shows fully washed Δ*ywdL* exosporium. The same amount of total protein (15 µg) was applied to each lane for comparison. Samples were treated with solubilisation buffer plus a 90°C incubation step to disrupt protein complexes.

The profile of proteins extracted at 90°C in solubilisation buffer from fully washed exosporium, and separated by SDS-PAGE is shown in Figure 8. Proteins were detected by silver staining. The protein profiles of wild-type and the Δ*ywdL* mutant appear generally similar, but there are minor differences – notably a stronger band between 14 and 20 kDa in the profile of Δ*ywdL* exosporium.

### YwdL is required for CaDPA-mediated germination

**Figure 9:**
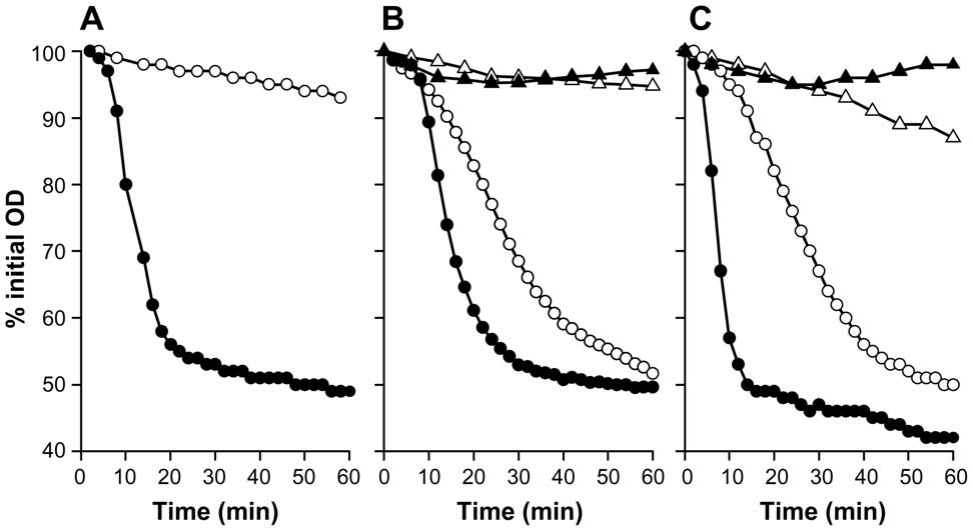
Germination of ΔywdL spores compared to wild-type. Germination in response to (A) CaDPA; (B) L-alanine (1 mM, with 10 mM OCDS as an inhibitor of alanine racemase) and (C) inosine (0.3 mM). Filled symbols represent *B. cereus* wild-type and open symbols the Δ*ywdL* mutant. Circles represent samples with germinant, and triangles samples without germinant. Data are representative of several experiments.

*B. cereus* Δ*ywdL* spores were analysed for germination defects in response to nutrient germinants L-alanine and inosine [25], which induce germination via receptor-mediated activation of germination-specific cortex lytic enzymes, and in response to the non-nutrient germinant CaDPA (calcium dipicolinate), which more directly stimulates activity of the cortex lytic enzyme CwlJ [26], [27]. In *B. subtilis*, YwdL is essential for spore germination initiated by exogenous CaDPA since it is required for the correct localisation of CwlJ in the spore coats [24]. A population of wild-type spores germinated completely in CaDPA within 25 min (Figure 9A), but germination was completely blocked for the Δ*ywdL* mutant. Germination rates in response to alanine and inosine were reduced (Fig. 9 B and C), as would be expected if cortex hydrolysis is slowed.

## Discussion

The results in this paper have emerged from our attempts to identify the proteins required to make up the structure of the exosporium. Two possible approaches to this problem include the comparison of wild-type with mutant structures and specific labelling of putative protein components whilst *in situ*; in this study we have tried both approaches. A *B. cereus* mutant with a transposon insertion inactivating the *ywdL* gene was isolated after enrichment for spores with reduced partition into hexadecane, reflecting reduced hydrophobicity. As described in previous work [13], [16], this has often been found to be due to altered or missing exosporium in mutant strains. The observation that the exosporium of a Δ*ywdL* mutant is more fragile than wild-type explains the enrichment of the Δ*ywdL* mutant from a transposon library. It is likely that Δ*ywdL* mutant spores lacking an intact exosporium were enriched, especially as the enrichment procedures were carried out on spores that had been stored frozen, then thawed (fresh spores of the Δ*ywdL* mutant showed similar partition into hexadecane compared to wild-type spores). These observations show that the lack of YwdL has an effect on gross exosporium structure. Moreover, immunogold labelling and Western blotting with anti-YwdL antibodies demonstrate that YwdL is present in the exosporium of *B. cereus* and *B. thuringiensis* strains (Figure 2, 3). It has also been previously identified in exosporium samples from *B. anthracis* by thin section immunogold labelling of whole *bclA* spores [22] and by tandem mass spectrometry of a tryptic digest of the exosporium [9]. Hence YwdL appears to be present in the exosporium of all *B. cereus sensu lato* members. In this study YwdL was detected as a band of apparent molecular weight of 43 kDa suggesting that it either forms a homotrimer or a higher order complex with other proteins (Figure 3). By examining whole spores rather than thin sections we have shown that YwdL is associated with the surface of the exosporium facing the interior of the spore; antibodies only labelled broken exosporium in which case they were able to access the interior surface (Figure 2). It has been speculated that antigens near the spore surface in *B. anthracis* might be partially obscured by the BclA-containing hairy nap [22]. This is not a likely explanation for the data in Figure 2, since the *B. thuringiensis* 4D11 strain has no nap on the exosporium (manuscript in preparation), and no antibody binding to YwdL was detected on the outer surface of intact spores of this strain despite the lack of nap. However, binding was detected on broken exosporium samples of *B. thuringiensis* 4D11 (Figure 2D). Moreover, YwdL is clearly bound to isolated exosporium as previously reported by Liu *et al.* [9] for unwashed *B. anthracis* exosporium fractions unless subjected to a rigorous washing regime (Figure 3); these washing conditions maintain the crystallinity of the exosporium basal layer but result in loss of YwdL (Figures 3 and 4).

We wished to test whether YwdL is a core structural protein of the exosporium. Comparison of washed exosporium fragments from wild-type and Δ*ywdL B. cereus* strains indicated no gross structural differences (Figure 4) or differences in the projected view of the crystalline exosporium basal layer (Figure 5). We conclude that YwdL is not required for either the assembly or the maintenance of the crystal lattice that makes up the basal layer of the exosporium. Moreover, digitally processed projection views of exosporium on intact spores not subjected to washing show features identical to those seen in Figure 5. This suggests that even when YwdL is bound in the unwashed wild-type exosporium it does not make a measurable contribution to the ordered density of the basal layer lattice. However, we cannot exclude the possibility that a full three dimensional analysis of the crystal structure embedded in vitreous ice might reveal subtle differences in density. Furthermore, the result of using digital image analysis techniques to analyse the exosporium structure results in the averaging out of any non-crystalline structural detail e.g. for BclA [5]. For this reason we also investigated the effect that YwdL might have on incorporation of the other known exosporium structural protein, BclA [21]. There was no measurable difference in BclA content in the Δ*ywdL* mutant (Figures 6 and 7A) or in glycoprotein content in general (Figure 7B).

The increased fragility of the exosporium in the mutant (Figure 1) suggests that YwdL has some more subtle effect on exosporium structure. It is this increased fragility, particularly in more aged spores that have been subjected to freeze-thaw cycling that most likely explains decreased partitioning in the hydrophobic phase in the initial mutant enrichment. In this case the effect has been due to exposure of the interior of the spore rather than any alteration in the surface properties of the exosporium itself. However, subtle differences between the protein composition of wild-type and Δ*ywdL* exosporium are also indicated in silver stain SDS-PAGE (Figure 8) in particular with more elevated levels of a protein visible at around 16 kDa. Thin section EM also indicates increased levels of electron-dense aggregates between the spore coat and exosporium in mutant spores (Figure 1C). These differences may be associated with an increased propensity for fractures to appear in the basal layer. Cross-linking effects could be important; in *B. subtilis*, YwdL is subject to crosslinking by a transglutaminase [28], and three lysine residues at the amino terminus have been identified as being involved in crosslinking with other proteins in the spore coat [29]. These residues are not present in YwdL from the *B. cereus* group. Nevertheless, in our study we have detected YwdL as a ∼48 kDa species (Figure 3) rather than the predicted monomeric 16 kDa size. This is consistent with the reported size of the major multimer of GerQ observed in *B. subtilis* spore coats [28], [29].

YwdL is highly conserved in *Bacilli*, though a homologue has not been identified in *Clostridia*. In this work, a *ΔywdL* mutation of *B. cereus* was identified on the basis of its apparent effect on the surface properties of spores, and its germination defective phenotype was also confirmed. This complements recent work on genes of the *cwlJ1-ywdL* operon, in which a Δ*ywdL* null mutant in *B. thuringiensis* was demonstrated to reduce germination rates in alanine and inosine [30], and data showing that both *ywdL* and *cwlJ1* in *B. anthracis* are required for germination and outgrowth in a rich medium [27]. YwdL is a spore coat protein that is required for the assembly of CwlJ-like cortex lytic enzymes into the spore coat of *B. subtilis*, essential for spore germination by Ca^2+^-DPA, but it does not appear to have a major effect on spore coat structure [24]. Our demonstration of an exosporium defect implies that it might have a wider morphogenetic role, affecting the stability of the exosporium.

## Materials and Methods

### Strains and plasmids used

*B. cereus* ATCC 10876, corresponding to *B. cereus* 569 UM20.1 used previously [13], was the parent strain for structural studies and for transposon mutagenesis with pLTV1. Strain AM1660 (*B. cereus ywdL*::Tn*917*LTV1) was isolated as described below. *Bacillus thuringiensis* Cry^−^ strain 4D11 [31], obtained from D. Ellar, was also used for exosporium studies.

### Phage transduction

Transduction was performed using phage CP51ts, as described previously [32].

### Media, growth and sporulation

Vegetative cells were grown in nutrient broth and spores prepared using CCY media as previously described [33], [12]. Spores were harvested when the culture contained >95% free spores followed by 10 washes in sterile ice-cold water to remove debris and vegetative cells. Washed spore pellets were resuspended in 50 mM Tris-HCl, 0.5 mM EDTA pH 7.5 at 20–50 mg ml^−1^ dry weight, and stored at −20°C.

### Exosporium isolation from B. cereus and B. thuringiensis

Exosporium fragments were isolated using the French press method as previously described [12] with minor alterations. Fragments were concentrated using a 10 kDa molecular weight cut off Vivaspin centrifugal concentrator (Sartorius) prior to further purification using an urografin density step gradient. Centrifugation at 145,000*× g* for 90 min was used to isolate exosporium fragments following dialysis to remove Urografin 370 (Schering). These ‘unwashed’ exosporium fragments were resuspended in ∼100 µl of 50 mM Tris-HCl, 0.5 mM EDTA pH 7.5 and stored at −20°C.

### Washing to remove loosely associated proteins from the exosporium

Exosporium fragments were washed with salt and detergent buffers as described by Todd *et al.* [12], however samples were washed for longer, each time incubating for 30 min on ice with gentle shaking prior to centrifugation at 145, 000*× g* for 2 hours. The final wash 5 was omitted. The “fully washed” pellet was resuspended at 1 mg ml^−1^ in 50 mM Tris-HCl 0.5 mM EDTA pH 7.5 and stored at −20°C.

### Sample preparation for SDS-PAGE

Protein concentration was determined using a BCA Protein Assay Kit (Pierce) according to the manufacturer's instructions. To fully disrupt the sample so that it enters the gel, 15 µg of isolated exosporia proteins were pelleted in a vacuum centrifuge (Eppendorf Concentrator 5301) and resuspended in ∼15 µl solubilisation buffer (50 mM CHES pH 9.8, 8 M urea, 2% SDS, 0.2 M DTT) then incubated at 90°C in a heating block for 20 min. 5 µl of NuPAGE LDS sample buffer was added prior to sample loading onto gels. For Western blotting 10 µg of isolated exosporium fragments were resuspended in 20 µl of NuPAGE LDS sample buffer plus NuPAGE Sample Reducing Agent (Invitrogen) and incubated at 40°C for 30 min prior to loading on the gel.

### SDS–PAGE

**S**amples were loaded on a 10% NuPAGE 1 mm Bis-Tris pre-cast gel (Invitrogen) using NuPAGE MES SDS Running buffer (Invitrogen). Gels were stained using Coomassie or Silver as previously described [12].

### Transfer of proteins onto PVDF

Proteins were transferred onto Hybond-P PVDF membrane (Amersham) as previously described [16] except that proteins were blotted at 60 V for 2 h using NuPAGE Transfer buffer (Invitrogen) containing 0.05% (w/v) SDS.

### Western blotting

Western blotting was performed using an ECL Plus Western Blotting Detection Kit (GE Healthcare) as previously described [16] using 2 µg ml^−1^ of monoclonal anti-BclA CR1 [16] and 1/5000 dilution of anti-mouse IgG-HRP linked whole antibody (GE Healthcare) for detection of BclA or 0.3 µg ml^−1^ polyclonal anti-YwdL antibodies [22] plus 1/5000 dilution of ECL anti-rabbit IgG-HRP linked whole antibody (GE Healthcare) for detection of YwdL.

### Total glycoprotein analysis of isolated exosporium

Glycoprotein staining was performed as previously reported [16] using 10 µg of fully disrupted exosporium (treated in solubilisation buffer and heated to 90°C) using an Amersham ECL Glycoprotein Detection Module (GE Healthcare).

### Immunogold labelling of proteins in whole spores

0.5 mg of whole water washed spores were resuspended in 1 ml of TBS-T buffer (50 mM Tris pH 8.0, 150 mM NaCl, 10% [v/v] Tween-20) plus 1% (w/v) BSA and mixed for 10 min with gentle shaking. Spores were centrifuged at 8,000*× g* for 5 min, washed twice in 1 ml TBS–T by centrifugation at 8,000*× g* for 5 min then incubated with 1 ml TBS-T+1% BSA+primary antibody (2 µg ml^−1^ or 0.3 µg ml^−1^ of monoclonal anti-BclA CR1 or polyclonal anti-YwdL antibodies respectively) for 1 hour with gentle shaking. Spores were centrifuged at 8,000*× g* for 5 min then washed 3 times as before in 1 ml TBS-T. Spores were incubated in 1 ml TBS-T+1% BSA+1/100 dilution of Goat Anti-mouse IgG; 10 nm Immunogold Conjugate (BB International) or Goat Anti-Rabbit IgG: 10 nm Immunogold Conjugate for 1 hour with gentle shaking. Spores were centrifuged at 8,000*× g* for 2 min, and then washed 3 times as before, centrifuging for 2 min instead of 5. Samples were resusupended in TBS buffer (50 mM Tris pH 8.0, 150 mM NaCl) and analysed by TEM. The whole procedure was performed at room temperature. Negative controls used were mutants lacking the protein of interest (Δ*ywdL*), or mouse anti-human B cell CD23 IgG primary antibodies (Dako) or anti-Goat IgG whole molecule produced in rabbit. An additional control replacing the primary antibody with buffer was also used.

### Spore sections

Ultra-thin sections (70–90 nm) were cut using a Reichert Ultracut E ultramicrotome and examined using a Phillips CM10 TEM as previously reported [13].

### Electron Microscopy

5 µl of spores at a concentration of ∼4 mg ml^−1^ and 3 µl of exosporium at ∼0.6 mg ml^−1^ were loaded onto carbon coated grids and stained with 0.75% (w/v) uranyl formate as previously described [5]. Samples were examined on a Phillips CM100 TEM at an accelerating voltage of 100 kV. Digital images were collected on a 1K×1K Gatan Multiscan 794 CCD camera. 2D projection maps were produced by merging 5 crystal images and processing using the MRC suite of programs [34]. Density maps were produced using the CCP4 crystallography programs [35].

### CaDPA germination Assays

Spores in 120 mM CaCl_2_ (100 µl) were added to a microplate well and pre-warmed at 25°C for 5 min. Pre-warmed 120 mM dipicolinic acid (100 µl, adjusted to pH 7 and containing 0.4% (w/v) gelatin) was added to give a final concentration of 60 mM CaDPA. The change in OD_490_ of the spore suspension was measured using a temperature controlled Wallac Victor2 1420 Multi-label counter.

### Measurement of heat, lysozyme and chemical resistance

Spore dilutions in water were incubated at 80 or 90°C for 15 min before plating on nutrient agar at 30°C to test for heat resistance. Counts were compared to those for unheated control samples. Assays of lysozyme resistance of spores and chemical resistance to ethanol were performed as previously described [16].

### Hexadecane partitioning assay

Hexadecane (0.1 ml) was added to 4 ml of spore suspension in distilled water at an OD_600_ of ≈1.0. The suspension was vortexed for 30 sec, and then left to stand for 15 min until the two phases had completely separated. 1 ml of the lower aqueous layer was removed with a sterile glass Pasteur pipette and the OD_600_ measured. The percentage transfer to the hexadecane layer was calculated as the percentage reduction in the OD of the aqueous spore suspension.

### Spore Germination

For nutrient germination, spores were activated by heating at 70°C for 30 min, placed on ice and used within 6 hours. Heat activated spores were equilibrated with germination buffer (10 mM Tris-HCl pH 8.4, 10 mM NaCl) in individual wells in microtitre trays. The suspensions were diluted in germination buffer to give a starting OD_490_ of approximately 0.5. For inosine germination, spores were incubated for 15 min at 37°C, then germination was initiated by the addition of pre-warmed inosine to 0.3 mM. For alanine germination, spores were pre-incubated at 30°C in germination buffer with O-carbamyl D-serine (10 mg ml^−1^), and germination initiated by the addition of pre-warmed L-alanine to 10 mM. Germination in the non-nutrient germinant CaDPA was measured using spores that had not been heat activated. Spores in 120 mM CaCl_2_ (100 µl) were pre-warmed at 25°C for 5 min. Pre-warmed 120 mM dipicolinic acid (100 µl, adjusted to pH 7 and containing 0.4% (w/v) gelatin) was added to give a final concentration of 60 mM CaDPA. The change in OD_490_ of the spore suspension was measured using a temperature controlled Wallac Victor2 1420 Multi-label counter, in which the samples were shaken for 10 seconds every two mins.
